# Hurdles to uptake of mesenchymal stem cells and their progenitors in therapeutic products

**DOI:** 10.1042/BCJ20190382

**Published:** 2020-09-17

**Authors:** Peter G. Childs, Stuart Reid, Manuel Salmeron-Sanchez, Matthew J. Dalby

**Affiliations:** 1 Centre for the Cellular Microenvironment, Division of Biomedical Engineering, School of Engineering, College of Science and Engineering, University of Glasgow, Glasgow G12 8QQ, U.K.; 2 Centre for the Cellular Microenvironment, SUPA Department of Biomedical Engineering, University of Strathclyde, Glasgow G1 1QE, U.K.; 3 Centre for the Cellular Microenvironment, Institute for Molecular, Cell and Systems Biology, College of Medical, Veterinary and Life Sciences, University of Glasgow, Glasgow G12 8QQ, U.K.

**Keywords:** biomaterials, cell growth, cell therapy, mesenchymal stem cell

## Abstract

Twenty-five years have passed since the first clinical trial utilising mesenchymal stomal/stem cells (MSCs) in 1995. In this time academic research has grown our understanding of MSC biochemistry and our ability to manipulate these cells *in vitro* using chemical, biomaterial, and mechanical methods*.* Research has been emboldened by the promise that MSCs can treat illness and repair damaged tissues through their capacity for immunomodulation and differentiation. Since 1995, 31 therapeutic products containing MSCs and/or progenitors have reached the market with the level of *in vitro* manipulation varying significantly. In this review, we summarise existing therapeutic products containing MSCs or mesenchymal progenitor cells and examine the challenges faced when developing new therapeutic products. Successful progression to clinical trial, and ultimately market, requires a thorough understanding of these hurdles at the earliest stages of *in vitro* pre-clinical development. It is beneficial to understand the health economic benefit for a new product and the reimbursement potential within various healthcare systems. Pre-clinical studies should be selected to demonstrate efficacy and safety for the specific clinical indication in humans, to avoid duplication of effort and minimise animal usage. Early consideration should also be given to manufacturing: how cell manipulation methods will integrate into highly controlled workflows and how they will be scaled up to produce clinically relevant quantities of cells. Finally, we summarise the main regulatory pathways for these clinical products, which can help shape early therapeutic design and testing.

## Introduction

As a multipotent cell type, mesenchymal stem (or stromal) cells (MSCs) have been a main source of focus within the field of regenerative medicine [1]. A set of criteria defining this cell population emerged in 2006 from the International Society for Cellular Therapy (ISCT) [2]. The ISCT criteria include: plastic adherence; tri-lineage differentiation potential (osteogenic, chondrogenic, and adipogenic); and a panel of surface markers which are expected (CD105, CD73, and CD90), and not expected (CD45, CD34, CD14, CD11b, CD79a, CD19, and HLA-DR) to be expressed. The ISCT criteria provide a highly beneficial benchmark to standardise studies, even when cell populations are sourced from different tissues. Distinct tissues such as bone marrow, peripheral blood, umbilical cord, and fat have all been shown to contain MSCs [3,4]. Comparative studies have demonstrated that tissue source can impact tri-lineage differentiation potential, along with other cell functions such as proliferation rate and cytokine expression [5,6]. Although cell source is important, there is therapeutic potential for all of these MSC populations, as demonstrated by comparative *in vivo* studies for osteogenic and chondrogenic repair where both were shown to have regenerative effect [7,8].

Even amongst MSC products, the therapeutic mode of action (MoA) will vary significantly based on clinical indication. From a European regulatory perspective, distinction is made between; somatic cell therapy medicinal products (sCTMPs), which illicit effect through pharmacological, immunological or metabolic means; and tissue-engineered products (TEPs), which aim to regenerate, repair or replace tissue [9]. In the field of regenerative medicine, MSCs are normally used due to their ability to differentiate into functional progenitor tissue types [1,10]. However, clinical efficacy may be determined by their longevity and ability to engraft. Typically MSCs have a transient and short engraftment duration which can limit their therapeutic efficacy [11]. Methods to increase the persistence of MSCs following implantation are, therefore, a key consideration for specific clinical applications. Biomaterial carriers can provide supportive environments for cells (e.g. injectable hydrogels and protein-based patches) and have shown the ability to retain 50–60% of implanted MSCs versus 10% of cells delivered via saline [12,13]. Pre-treatment of cells (with hypoxia or cytokines) can prepare them for ischemic environments [14] and pharmacological treatment can minimise lineage commitment (e.g. inhibition of the Wnt pathway to maintain MSC multipotency) [15] allowing improved persistence upon implantation. As well as increasing longevity, it has been demonstrated that biomaterials can support MSC viability and drive differentiation via cell–material interactions [16,17].

In terms of immunological MoAs, MSC can interact with immune cells, including T-lymphocytes and dendritic cells. This capacity increases opportunities for allogeneic transplant procedures [18,19] with MSCs acting as a suppressive ‘drug’. The mechanism involves cell-to-cell contact and also the MSC secretome, which includes key factors such as: transforming growth factor beta 1 (TGFb1), hepatocyte growth factor (HGF), C-X-C motif chemokine ligand (CXCL)-10, and CXCL-12 [20,21]. The paracrine impact of MSCs contrasts from the direct replacement of damaged tissue and allows treatment of conditions such as graft-versus-host disease (e.g. as a result of marrow transplantation) [22] or to support islet transplantation [23]. Indeed, such immunomodulatory and anti-inflammatory properties are helping MSCs to find applications in cardiac, hepatic, and even neuronal regenerative approaches [24–29]. As the use of therapeutic MSCs grows it has become important to consider how cell expansion will be achieved, and if a naïve phenotype can be maintained. For some therapeutic purposes, it may be desirable to manipulate MSC phenotype, or to even differentiate them during this process. To successfully provide a therapy or build a business, provision of billions, or even trillions (depending on dosage), of MSCs is required [30]. At the same time maintaining the desired phenotype is central to the reproducibility of the final therapy.

In this review, we will examine key considerations when seeking to translate MSC/progenitor therapies from the academic laboratory to clinic. We will discuss: control of MSC phenotype; scale-up of cell culture; and the impact on commercial, clinical, and regulatory viability.

## *In vitro* manipulation of MSCs

To be specific, in this review, and in general clinical use, when we describe MSCs we are discussing the whole adherent population of the stroma that will include stem and progenitor cells. They are often described as mesenchymal stem cells, but, strictly, the stem cells are a clonogenic population of stromal cells able to recreate cartilage, bone, haematopoiesis-supporting stroma [31,32]. The stem cells are typically purified using CD markers and magnet-activated cell sorting/flow sorting. However, selection of CD purified populations significantly reduces cell number. The extent of this reduction is dependent on the specific markers, pre-purification steps and source tissue used [33,34]. Therefore, a pragmatic decision is typically made to use the whole unselected stromal population.

Although the ISCT criteria are beneficial in terms of quality control, they are not the only consideration from a commercial perspective. As the number of MSC products grow it will be crucial for new therapeutics to distinguish themselves from competitor products [30]. This could include targeted clinical functionality or novel product/process intellectual property. As a result, many products will seek to supplement the standard ISCT criteria with additional phenotypic markers relating to the intended clinical use. Some clinical indications will benefit from a naïve, immunomodulatory, MSC phenotype (e.g. graft-versus-host disease), whilst others may benefit from MSCs showing markers of early osteogenic differentiation (e.g. fracture repair) [35]. As an example, Stro-1 and CD271 have both been identified as MSC markers but are not expressed across all tissues [34]. Stro-1 positive MSCs have been linked with cardiac regeneration [36] whilst Stro-1 negative MSCs support haematopoietic stem cell engraftment [37]. Stro-1 expression can both increase or decrease throughout the culture, a key consideration for cell expansion [34]. CD271 can be co-expressed with other MSC markers and has been linked with improved cartilage repair when compared with CD271 negative MSCs [34,38]. Before reviewing current MSC therapies, we will briefly discuss materials and methods to manipulate MSC phenotype, including the maintenance of potency, a key consideration during cell expansion and other manufacturing processes.

Classically, chemical differentiation via soluble factors has been the go-to method of controlling MSC phenotype through use of specific growth factors or chemically defined media [39]. However, these methods can have their limitations of specificity with typical osteogenic reagents (e.g. dexamethasone, ascorbic acid, and β-glycerophosphate) also stimulating expression of adipogenic markers [40,41]. As an alternative, materials have proven useful tools in manipulating and understanding MSC growth and differentiation mechanisms. It is now understood that material chemistry, mechanical environment, and topography can each control MSC fate [17]. The ‘materials’ surrounding a cell, the extracellular matrix (ECM), are formed of proteins containing a rich milieu of biological factors such as adhesion peptide sequences (e.g. arg-gly-asp or RGD) and growth factors. The ECM also provides physical stimulus through varying stiffness, topography, and chemistry [35]. Cells more readily interact with this environment than the non-proteaceous man-made environments of culture plastics or inert biomaterials [42]. However, when synthetic materials are placed into culture media, or into the body, they absorb ECM proteins, and cells can then ligate via integrins to peptide motifs such as RGD [42]. This ligation is of central importance to MSC phenotype. As the cells adhere, integrins gather into focal adhesions and signalling proteins such as focal adhesion kinase (FAK) associate with the growing adhesion complex [43,44]. Stress fibres terminate at adhesions and signalling proteins drive actin contraction via biochemical mediators such as mitogen-activated proteins kinases (MAPKs) and extracellular signal-related kinase 1/2 (ERK 1/2) [35,45]. In fact, for MSCs, the size of the adhesions can be predictive of phenotype with adipocytes having very small adhesions (<1 µm length) and osteoblasts large, mature, adhesions (>5 µm length); fibroblasts and MSCs have intermediate-sized adhesions as will be discussed [35,46].

In considering MSC-material interactions, research first focussed on differentiation and key papers highlighted the role of intracellular tension in determining cell fate [17]. Use of cell containment in small, adhesion motif-rich fibronectin islands revealed that constraining MSCs so that they could not spread resulted in adipogenesis, while promoting spreading in larger islands drove an osteogenic response. Probing of mechanism revealed the roles of cytoskeletal tension mediated via RhoA kinase (ROCK) which controls actin-myosin interaction and cell contraction. This was illustrated by ROCK overexpressing cells in constrained morphologies developing into osteoblasts while inhibiting ROCK in well spread MSCs pushed differentiation towards adipogenesis [47]. A subsequent study used fibronectin patterns of similar size but differing shapes — stars and flowers. The rounded flower shapes were less amenable to cell adhesion and to the development of resultant intracellular tension than the sharp star shapes, which promoted adhesion and tension. Despite being the same size, MSCs differentiated preferentially to osteoblasts on the stars and adipocytes on the flowers and this, again, was seen to be ROCK dependant [45], helping to demonstrate that intracellular tension is important in MSC differentiation.

Stiffness has also been shown to direct adipogenesis and osteogenesis, with low stiffness environments directing adipogenesis while stiffer environments direct osteogenesis [48]. This is because MSCs in low stiffness niches share their intracellular tension with the material as they deform it resulting in a lower apparent cytoskeletal contraction. However, in stiff environments, MSCs experience all the cytoskeletal tension, hence driving osteogenesis [16]. Differentiation is morphology independent and tension dependant, as evidenced by stiffness driven osteogenesis not having concomitant enhanced cell spreading in 3D stiff matrices [49]. Nanoscale topographical patterns that drive osteogenic MSC differentiation also enhance adhesion and intracellular tension [50,51].

More recently, MSC interactions have been studied with materials incorporating controlled viscous, as well as elastic, properties [52]. These materials have again highlighted new methods to control MSC phenotype through modification of adhesive ligand mobility and introduction of time dependant material changes (e.g. controlled stress relaxation) [53]. Quicker stress relaxation aids material remodelling and supports the formation of relevant ECM to support differentiated tissues such as cartilage and bone [54,55].

The adipose and osteoblast differentiation mechanisms of MSCs have been the subject of intense study. However, the expansion of MSCs with maintained phenotype is much less well understood. Again, papers have emerged showing roles for topography [56], chemical patterning [57], and stiffness [58,59].

**Figure 1.**
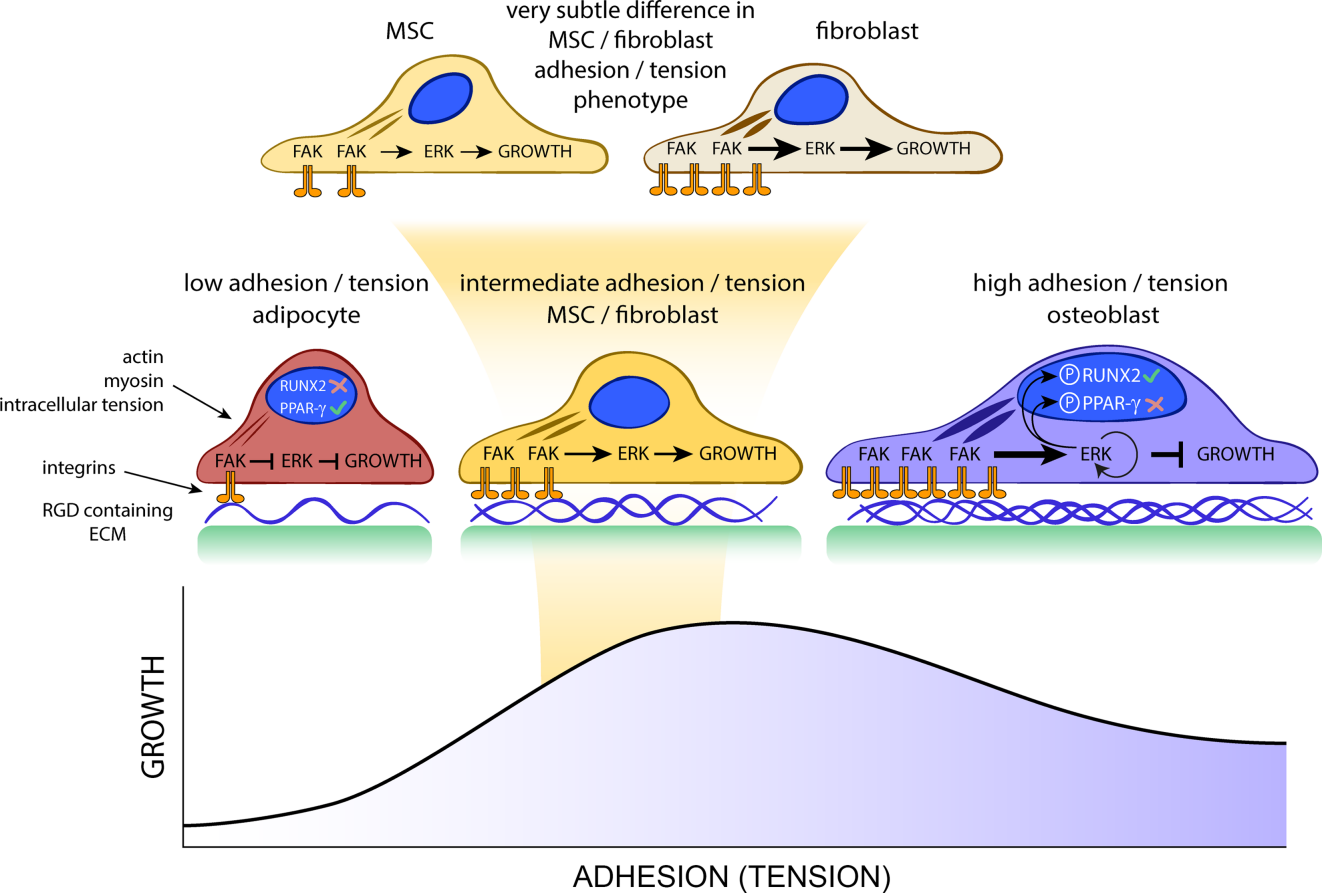
Correlation between stem cell adhesion and growth. Low-adhesion phenotypes, such as adipocytes, have limited cell adhesion, low intracellular tension, low ERK 1/2 activation by FAK, and therefore, growth is very slow. High-adhesion phenotypes, such as osteoblasts, establish large adhesions driving increased intracellular tension. FAK activates ERK1/2 and a negative feedback loop limits growth in this phenotype. Fast-growing mesenchymal stem cells (MSCs) and fibroblasts represent intermediate adhesion-tension phenotypes, with integrin and FAK clustering, but with the subtle difference that MSCs have lower intracellular tension than fibroblasts. The thickness of the lines between the integrins and the nucleus represents the amount of cytoskeletal tension generated through adhesion. Image adapted from Dalby et al. [35].

Using these material surfaces, various mechanisms have been revealed. MSCs appear similar to fibroblasts, indeed they were identified as fibroblast colony-forming units due to their fibroblastic morphology, clonogenic growth, and ability to differentiate [60]. However, it has been seen that while morphologically similar, MSCs have a slightly lower tension phenotype to fibroblasts; both being in the middle of adipocytes (low) and osteoblasts (high) in terms of intracellular tension [50]. Both fibroblasts and MSCs are fast-growing cells compared with adipocytes, where low adhesion results in low levels of ERK 1/2 activation, and osteoblasts, where very high levels of adhesion drive negative feedback on ERK 1/2 causing activation of bone-related transcription factors at the cost of proliferation [61,62]. While the growth rate is similar to fibroblasts, MSCs maintaining a naive phenotype appear to have subtle changes in cell cycle regulation. For example, cyclin dependant kinase 6 (CDK6), which is a positive regulator of cell cycle progression and linked with reduced sensitivity to the osteogenic growth factor bone morphogenetic protein 2 (BMP2) is up-regulated in MSC populations maintaining their phenotype on nanotopography [62]. These points are developed in Figure 1.

The scale up to very large quantities of clinically useful MSCs, as will be discussed in this review, is an area of ongoing development. Methods to maintain cell phenotype during this process may be of significant value, whether MSC naivety or maturation is required. However, these methods and materials must be compatible with industrially relevant cell expansion systems.

## Clinical application of MSCs

To understand the relevancy of *in vitro* MSC manipulation it is beneficial to examine the current level of clinical development and existing uses for MSCs in the treatment of disease. Although our understanding of *in vitro* MSC phenotype has grown rapidly in preceding decades, this has not directly led to widespread clinical application. Ever since the first clinical trial of MSCs in 1995 [63], their use to repair damaged tissues has been highly anticipated.

In 2018, the ISCT published a list of global tissue, gene, and cellular medicinal products, of which around 41 are cell based with marketing approval within one or more regulatory region [64]. However, this list does not include medical devices containing cells. Globally, as of 2019, 31 therapeutic products have reached market containing MSCs or mesenchymal progenitor cells (e.g. osteoblasts, chondrocytes) [65]. This is despite thousands of MSC focussed academic publications now being produced each year [66]. Several of these products are demineralised bone matrix which have retained their MSC and osteoprogenitor populations during processing [67], but these are regulated separately to biologic products.

The regulatory classification for these two types of MSC product varies between regions. These bone matrix products are largely marketed in the U.S.A. where cultured, manipulated, or processed cells, and cells used in a heterologous manner, are often classified as ‘351 products’ under human cells, tissues, and cellular and tissue-based product (HCT/P) regulation [68]. However, allogeneic tissue which is used in a homologous manner (e.g. demineralised bone matrix with cells used for bone grafting) is covered by section 361 of HCT/P regulation and subject to a lighter regulatory assessment. In the U.S.A., cell therapies which are covered by section 351 are classed as biologic products and subject to full premarket review, i.e. clinical trials and biologics licencing similar to devices or drugs [69]. A list of products which contain MSCs (previously and currently marketed) is shown in Table 1, including details of their clinical application, dosage and cost. Similarly, Table 2 lists previous and current products which use mesenchymal progenitor cells (e.g. osteoblasts or chondrocytes). It is notable that there are no products currently on the market which contain MSCs that have been pre-differentiated *in vitro.* Although, several, such as Bone Therapeutics’ Allob (osteogenically stimulated allogeneic bone marrow MSCs), are undergoing clinical trial [70].

**Table 1.** Previous/currently marketed clinical products containing MSCs

Therapy name	Product description	Clinical indications	Release	Market region	Dose	Cost/unit or dose
Osteocel	Allogeneic Bone marrow MSCs	Orthopaedic repair	2005	U.S.A.	3 m cells/cc [71]	$460/cc [71]
AlloStem	Allogeneic adipose MSCs	Orthopaedic repair	2010	U.S.A.	66 255 cells/cc [71]	$540/cc [71]
CardioRel	Autologous MSCs	Myocardial infarction	2010	India	N/A	N/A
Queencell	Autologous adipose cells	Subcutaneous tissue defect	2010	South Korea	70 m cells [72]	N/A
Cartistem	Umbilical cord-blood MSCs	Cartilage defects of the knee (osteoarthritis)	2011	South Korea	5 m cells/ml [73]	$19 000 [74]
Cellgram- AMI	Autologous bone marrow MSCs	Acute myocardial infarction	2011	South Korea	50–90 m cells [75]	$15 000 [76]
Grafix	Allo. placental membrane, incl. MSCs	Acute/chronic wounds	2011	U.S.A.	N/A	N/A
Cellentra VCBM	Allogeneic MSCs in bone matrix	Orthopaedic repair	2012	U.S.A.	>250 k cells/cc [71]	$620/cc [71]
Cupistem	Autologous adipose MSCs	Crohn's fistula	2012	South Korea	160 m cells [77]	$3000–$5000 [74]
Prochymal	Allogeneic MSCs	Acute graft vs host disease	2012	New Zealand/Canada	2 m cells/kg (10 doses) [78]	$200 000 [79]
HiQCell	Autologous adipose stromal vascular fraction	Osteoarthritis/tendonitis	2013	Australia	N/A	AUD 1000 [80]
Trinity ELITE	Allogeneic MSCs in bone matrix	Orthopaedic repair	2013	U.S.A.	>500 k cells/cc [81]	N/A
Map3	Allogeneic demineralised matrix and multipotent cells	Orthopaedic repair	2014	U.S.A.	N/A	N/A
Neuronata-R	Autologous bone marrow MSCs	Amyotrophic Lateral Sclerosis	2014	South Korea	1 m cells/kg (every 2 weeks) [82]	$55 136 p/a [83]
OvationOS	Allogeneic MSCs in bone matrix	Orthopaedic repair	2014	U.S.A.	>400 k cells/cc [71]	$2700/cc [71]
Temcell HS	Allogeneic marrow MSCs	Acute graft vs host disease	2015	Japan	2 m cells/kg (12 doses) [84]	$113 000–$170 000
Stempeucel	Allogeneic MSCs	Critical limb ischemia	2016	India	2 m cells/kg [85]	₹150 000 [86]
Alofisel	Allogeneic adipose MSCs	Perianal fistulas in Crohn's disease	2018	Europe	120 m cells [87]	£54 000 [88]
Stemirac	Autologous bone marrow MSCs	Spinal cord injury	2018	Japan	50–200 m cells [89]	$135 000 [90]
Trinity Evolution	Allogeneic MSCs/progenitors in bone matrix	Orthopaedic repair	2019	U.S.A.	>250 k cells/ cc [71]	$540/cc [71]

**Table 2.** Previous/currently marketed products containing MSC progenitors

Therapy name	Product description	Clinical indications	Release	Market region	Dose	Cost
Carticel	Autologous chondrocytes	Articular Cartilage repair	1997	U.S.A./EU	0.6–3.3 m cells [91]	$13–15 k [91]
Chondron	Autologous chondrocytes	Focal cartilage defect	2001	South Korea/India	12–72 m cells [92]	₹3–400 k [93]
DeNovo NT	Allogeneic cartilage with chondrocytes	Articular Cartilage repair	2007	U.S.A.	2.5 cm^2^ fill [94]	$4–5 k [95]
Chondro- celect	Autologous chondrocytes	Articular Cartilage repair	2009	EU	4 m cells [96]	£18 301 [96]
Ossron	Autologous osteoblasts	Focal bone formation	2009	South Korea	12–72 m cells [92]	₹3–400 k [93]
JACC	Autologous chondrocytes in collagen gel	Articular Cartilage repair	2012	Japan	45 k cells [97]	N/A
MACI	Autologous chondrocytes on porcine membrane	Cartilage defects of the knee	2016	U.S.A./Europe	500 k cells/cm^2^ implant [96]	£16 226 [96]
Ortho-ACI	Autologous chondrocytes	Cartilage lesion of the knee, patella and ankle	2017	Australia	4–10 m cells [98]	AUD 6500–10 000 [99]
Spherox	Autologous chondrocytes (spheroids)	Cartilage defects of the knee (<10 cm^2^)	2017	Europe	Up to 100 spheroids [100]	£10 000 [101]
Ossgrow	Autologous osteoblasts	Avascular necrosis of the hip	2017	India	48 m cells [102]	₹140 000 [103]
Cartigrow	Autologous chondrocytes	Cartilage defects of the joints	2017	India	12 m cells [104]	₹140 000 [103]

As can be seen from Table 1, price varies by orders of magnitude between products, which is not readily explained by the variance in dosage. It is notable that products with lower costs, such as AlloStem, Cellentra, and Trinity Evolution, all fall under section 361 of HCT/P regulation where market access is easier to obtain. Another key factor to note is the lack of products incorporating differentiated MSCs. This is despite products existing which focus on repair of tissues such as cartilage and bone.

Although many of these marketed products involve *in vitro* expansion of the cells, few seem to involve structured carrier materials. By this we mean using cells and a scaffolding material such as a gel, microparticle, or polymer; an area of significant ongoing academic development [105,106]. Crucially, carrier materials may assist the efficacy and longevity of cells once implanted. In terms of marketed products the exceptions are MACI (matrix-induced autologous chondrocyte implantation), which includes a carrier membrane [107], and Spherox, which forms spheroids [100]. These strategies are designed to deliver and retain cells at the local site. In the case of Spherox (also named chondrospheres), the specific use of spheroids increases the cells’ ability to produce key constituents of cartilage (collagen II and glycosaminoglycans) over prolonged implantation durations (up to 24 weeks), when assessed in murine models. As a result, newly synthesised cartilage was produced and integrated with the surrounding native matrix [108].

**Figure 2.**
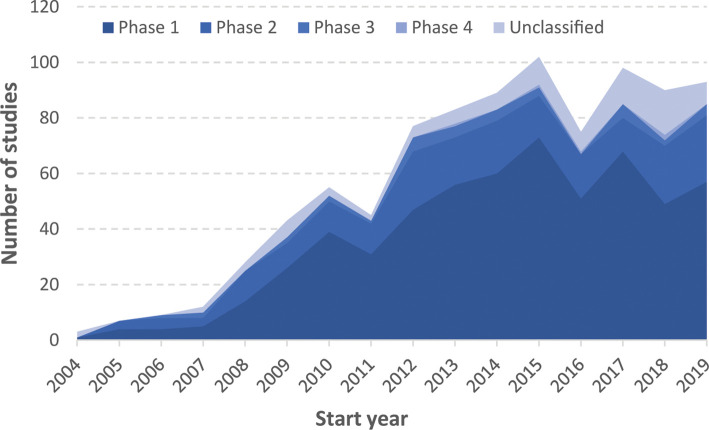
Clinicaltrials.gov entries for interventional studies starting in the years 2004–2019. The data show the number of clinical trials using mesenchymal stem/stromal cells which started each year. The chart separates the trials each year by trial phase, where information is available in the database. Data collected November 2019.

Although relatively few structured (e.g. with biomaterials) MSC products have reached market it is apparent that there is a significant pipeline of therapeutic products currently progressing through clinical trial. Figure 2 shows the growth of MSC focussed clinical trials over the past 15 years and includes 909 studies from 1st January 2004 [109]. Figures include trials listed on clinicaltrials.gov which reference the use of mesenchymal stem cells or mesenchymal stromal cells. This includes studies using cultured MSCs and also MSCs as part of bulk tissues (e.g. bone marrow aspirate) but excludes observational studies (i.e. studies without a defined MSC-based intervention). As can be seen, there was a rapid growth in the number of experimental MSC therapies during this time frame which appears to have now stabilised at a level of roughly 50–70 new phase I studies per year. Also clear from this data are the high levels of attrition through each clinical trial phase, with drastically fewer experimental therapies being tested in phase III studies (less than 5 per year). Although, it is not apparent what the reason for attrition is as there are many factors which can contribute to failure to reach the market. Factors such as insufficient efficacy, inability to scale manufacturing, and high reimbursement prices can factor into the health economic assessment of product viability and ultimately cease therapeutic development.

## Health economics of cell therapies

Although seemingly a commercial concern, health economics are a key factor dictating successful therapeutic translation and are ideally considered during product conceptualisation [110,111]. A clear clinical need must be established, ideally where current clinical solutions are limited or there is an opportunity to decrease the overall cost of treatment (e.g. reduced surgical time, hospital admission times, or increases in quality of life) [112]. An assessment of the innovation headroom is then required to demonstrate that the expected therapeutic benefits will exceed the expected cost of treatment. There are many frameworks used to decide on uptake of new therapies depending on region, including cost-benefit analysis, cost-consequence analysis, and budget impact analysis [112].

As example, in the U.K. the National Institute for Health and Care Excellence (NICE) assesses the cost effectiveness of new therapies and makes recommendations on their adoption within the NHS. Therapies are scored based on their ability to provide patients with increased quality-adjusted life years (QALYs). Although subject to many additional factors, NICE typically assumes that a cost lower than £20 000 per QALY gained is cost effective, a value determined by independent committee [113]. However, even if a new treatment is determined cost effective for an individual patient, the overall budgetary impact (based on the number of anticipated patients) may pose limitations dependant on overall national healthcare budgets [114].

Early understanding of target pricing can also be highly beneficial to shape product development at the earliest pre-clinical stages. Cellular products are typically highly engineered and manufacturing intensive, it is essential to ensure that healthcare payers will be willing to cover potentially high reimbursement costs for the target indication [115]. For this reason, new commercial models are being defined, including deferred/instalment payment for therapies, based on continued delivery of patient outcomes [116]. In these arrangements, it would be increasingly important for the therapy to establish long-lasting patient benefits.

As shown in Tables 1 and 2, products vary drastically in price. The South Korean autologous MSC therapy, Cellgram-AMI, costs in the region of $15 000 per treatment. The clinical target for this product is damage due to myocardial infarction and the clinical endpoint measured was left ventricular ejection fraction, 6 months after treatment. For one dose a 6% improvement in ejection fraction resulted [117].

In some cases, high reimbursement costs have not been matched by sufficient improvement in patient outcomes, with some of the first therapeutics to reach market subsequently failing commercially. Key examples include autologous chondrocyte implants (ACIs), e.g. ChondroCelect and MACI (matrix-induced ACI). Both achieved market approval in Europe but have since failed to secure national reimbursement from key countries. This has led to them being withdrawn from the market [118]. Although not an MSC therapy, Provenge illustrates this challenge. Provenge is an autologous immunotherapy for the treatment of prostate cancer which aimed to secure reimbursement of $93 000 per dose. However, the demonstrated patient benefit equated to between 2 and 4 months increase in survival, a level which could only expect to justify costs of $30 000 in the U.S.A. [110,118]. As such, the therapy has failed to achieve reimbursement in the U.S.A. or Europe.

The ability for new therapies to achieve reimbursement largely relates to the balance of efficacy and production/testing cost. At the earliest stages of development, a key focus should be to define the target clinical indication and relevant outcome measures via broad engagement with clinicians [119]. Once known this can be used to select appropriate *in vivo* models for efficacy testing. Regarding the product itself, it is crucial to lock a variety of product parameters early on in development (e.g. cell source, administration method, carrier materials) based on commercial and clinical appropriateness. This can reduce repetition when progressing through *in vivo* testing. Product parameters such as autologous vs allogeneic starting material, or the ability to cryopreserve cells can lead to drastic impacts on the subsequent commercial model and production cost [120,121].

## Pre-clinical testing of cellular products

Beyond initial *in vitro* evidence of cell activity and phenotype, *in vivo* models should show both efficacy and safety of the final therapeutic. Animal models should reproduce, as closely as possible, the condition being targeted in humans for data to have maximum relevance [122]. However, the ability to do so is often determined by the availability of such models, either academically or through contract research organisations (CROs).

At the earliest stages, *in vivo* efficacy testing can often follow an iterative process whilst production parameters (e.g. culture conditions, administration methodology) are honed for the specific therapeutic target. As such, an early consideration of reagents and processes which can be carried over into good manufacturing practice (GMP) manufacture can minimise the need for later *in vivo* re-testing and minimise the potential for subsequent failure [123]. In addition, the levels of efficacy demonstrated *in vivo* should aim beyond simple statistical significance and should demonstrate clinical significance [124]. This is particularly relevant when comparing the new treatment to controls simulating the current standard of care. The study design is a key consideration and efforts should be made to ensure studies are sufficiently powered and follow protocols which maximise reproducibility [125].

If efficacy is demonstrated in small animal models, such as rodents, then many therapeutics progress onto larger animal models. This is often the route chosen where there is uncertainty over the ability to scale the therapy (e.g. in the repair of larger volumes of tissue) [126]. However, the necessity of large animal studies is a matter which will vary based on: clinical trial regulator, the condition being treated, and the prior clinical use of similar cellular products [127]. The importance of this type of study for human translation is still a matter of debate [128]. Indeed, when performing these models it is debatable if the best route is to test the human cell line with immunomodulation of the animal, or to produce an equivalent cell line derived from the species being tested. The relevancy of either scenario to final human use is questionable. For this reason, there is growing interest in humanised *in vitro* or *ex vivo* models [129–131].

Beyond efficacy, the safety of any new cellular product must be demonstrated *in vivo* as part of any application to perform clinical trials. Although it may be possible to collect indicators of safety during initial studies, a comprehensive safety assessment of the final therapy formulation will be required through pivotal safety testing [132]. This is usually carried out on GMP manufactured cells, or GMP-like cells to demonstrate equivalence with the final product [133].

The design of pivotal safety studies will be specific to the therapy and in the European Union guidance is provided that a risk-based approach should be adopted [134]. This aims to identify potential adverse outcomes and then design the pivotal studies to examine their likelihood. For MSCs there are number of common risks to consider including but not limited to: tumorgenicity, immunogenicity, chromosomal instability, and unsafe biodistribution profiles [22,135–137]. From a regulatory perspective, it will be required to justify the model's appropriateness, both in terms of simulated condition and duration. Furthermore, it is typical to carry out these studies to good laboratory practice (GLP) standard, including data analysis [133]. The availability of GLP models can be a barrier to progression and may require co-development of a new model with a commercial test house.

## Transition to GMP manufacture

It is important not to underestimate the complexity of moving academic protocols into an industrial therapeutic manufacturing facility. This can include challenges related to reagents, specialised equipment, poorly defined user-dependant steps and finally, scalability [115]. Technically there can also be difficulties characterising the cells and linking this to their clinical potency. This requires a thorough understanding of their intended MoA. Finally, therapeutic cells need to be measurably consistent from batch to batch and upon delivery into clinic [138].

Manufacturing strategies vary depending on the specific product and clinical delivery route. Autologous products may require cellular manipulation close to clinic, whereas allogeneic products benefit from the ability to manufacture at a centralised facility. The number of manufacturing sites also becomes a consideration when looking to supply larger numbers of doses or supply across multiple regions [139]. However, for any steps considered ‘substantial manipulation’ there is the general requirement that these occur in a facility with GMP accreditation and within a cleanroom to ensure product safety. The specific grade of clean room will depend on the culture systems used, i.e. open (e.g. a culture flask requiring opening for media exchange within a flow cabinet) or closed (e.g. bag culture with automated media exchange on benchtop) [140,141].

With the growth of therapeutic candidates, the number of industrial cell manufacturing facilities has also increased [142]. Organisations such as the U.K.’s Cell and Gene Therapy Catapult provide quick connections and access to national GMP manufacturing hubs and guidance for the engagement with industrial manufacturing partners. Similar networks also exist at the European level including the European Society for Gene and Cell Therapy and the European Commission funded Restore initiative.

One of the major differences between therapeutic manufacture and academic development is the requirement to use GMP validated reagents and cultureware. For many *in vitro* cell manipulation protocols the use of novel reagents or biomaterials may pose a barrier to GMP manufacture, this is particularly true for cell culture media [143]. It is common for academic cell culture to use animal products such as porcine trypsin or basal media supplementation with foetal calf serum (FCS). However, this introduces potential for disease transmission, batch variability, and insufficient supply [144]. Efforts should be made to find xeno-free reagents and supplements which can support the desired levels of cell growth and end yields. Commonly this involves replacement of FCS with human serum (viral tested), other blood-derived supplements (e.g. human platelet lysate) or commercial serum-free formulations [143,145]. Alterations of culture conditions can lead to significant changes in cell phenotype and clinical functionality. For this reason, early introduction of GMP validated reagents can increase the relevance of pre-clinical test data.

Another raw material which is crucial to secure are the cells themselves. For allogeneic MSC supply it will be necessary to establish a procurement strategy which encompasses donor consent and confidentiality, along with a banking strategy involving a master donor cell bank and working bank of therapeutic doses [138,146]. Quality control of the donor material is required, both in terms of safety, identity, and expected potency. This could include testing for MSC surface markers, infectious agents, and genetic abnormalities [147]. In terms of *in vitro* manipulation of MSCs, it may be advantageous to pre-screen cells for their differentiation potential or immunomodulatory capacity to ensure higher yields of the desired cell population at the end of production.

At the end of production, assessment of the final product will be required to demonstrate comparability with set release criteria. Both product safety (e.g. no increases in tumorgenicity) and correct cellular identity will need to be demonstrated [147]. The cellular identity will be highly individualised to the specific therapy and assessment may involve flow cytometry for specific surface markers or assessment of secreted proteins. It can also be these release criteria which form the central claims for intellectual property protection. For example, Bone Therapeutics’ MSC derived products are defined by MSC surface markers, CD105, CD90, CD73, and CD34, and this identity, along with specified growth factors used during production, are central to their patent protection [148].

In terms of long-term potential, scalability can be a major barrier if phenotype is likely to change during culture. Scalability involves *in vitro* MSC expansion to produce clinically relevant quantities of cells to perform trials and then to support a company selling a product into multiple centres. As discussed previously, the cost is a major factor in therapeutic adoption and cell yield during manufacturing is a critical factor driving the end cost [149]. For this reason, there is a balance to be struck between higher cell yields and maintenance of the cell identity at the end of manufacture [150].

There already exists many reviews exploring the scaling of cell manufacture [151–153]. However, the first step for smaller clinical trials typically involves transition to multi-layer cell stacks. These have capacities in the region of 10 million cells per layer and are regularly used in GMP culture conditions. These planar cell culture methods are suitable for small clinical trials but become increasingly labour intensive when moving to larger trials where billions of cells may be required [151]. Larger scale culture strategies include bioreactors which can provide increased culture area through the use of hollow fibres or microcarriers [154,155]. Significant work has already been carried out by groups such as the Cell Technologies Research Group at Aston University to understand how microcarrier culture differs from, and could replace, planar, flask-based, culture. This has included studies to examine: serum-free/human serum culture media, optimum rotation speed of spinner flasks, metabolite production, and the choice of microcarriers [30,156–159]. For MSC expansion these types of vessel have been validated up to culture volumes of 2 l; equivalent to 760 million MSCs per batch [160]. In most of these studies, it has been demonstrated that the ISCT criteria for MSCs are met, even following expansion [157,161]. This crucial translational research increases confidence that MSC therapies can continue to be scaled and gain further industrial relevance.

**Figure 3.**
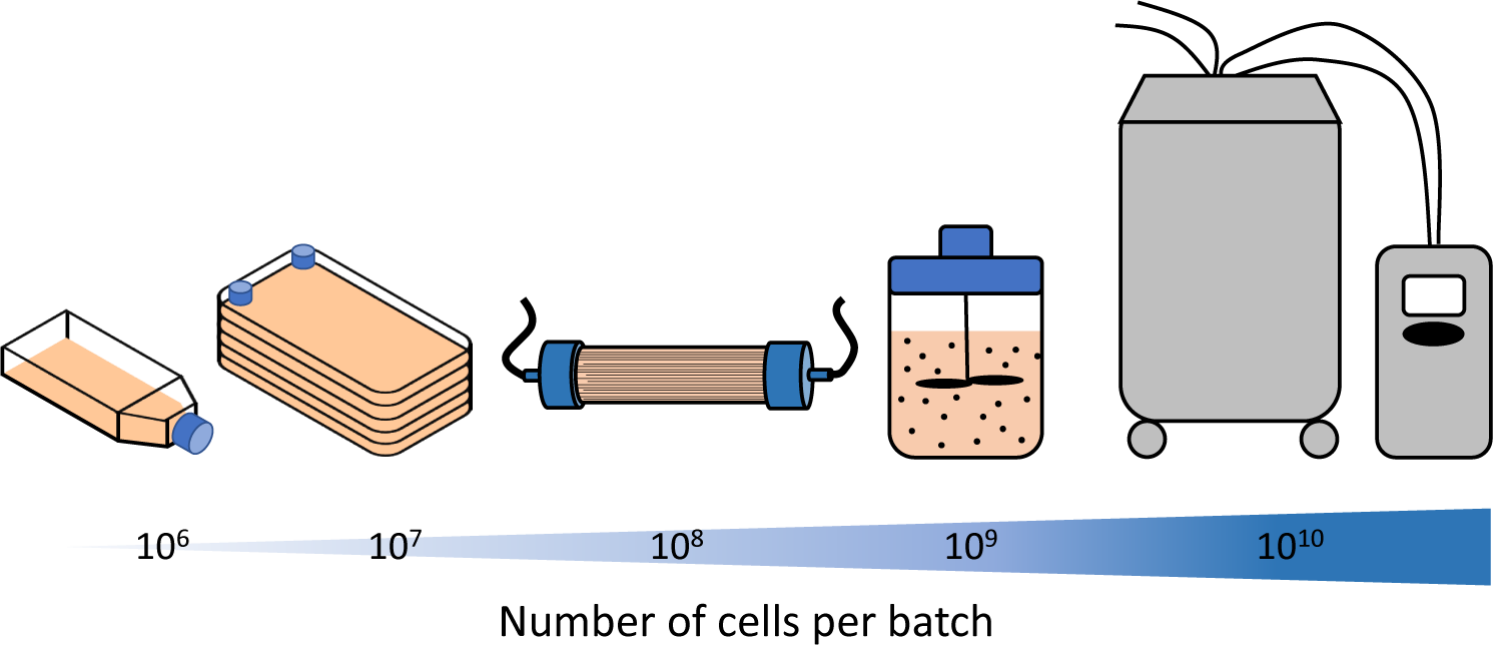
Potential culture technologies for cell manufacture. Monolayer cell culture methods start at the scale of standard tissue culture flasks with yields of several million cells per flask. To supply clinical trials it is necessary to consider the expansion of cells in larger culture vessels, such as multi-layer cell stacks or hollow fibre systems providing increased surface area for growth. Beyond this, microcarrier based culture via small and industrial scale stirred tank systems may present a route to supplying billions of cells per batch.

Many commercial systems are being developed, including with built in automation (Figure 3). The Cell and Gene Therapy Catapult have carried out systematic comparisons of planar and automated hollow fibre culture systems which demonstrate the labour and cost benefits of these commercial hollow fibre systems [162]. Beyond the supply of clinical trials, where tens of billions of cells are required, there will be need to go further than these existing technologies, either finding ways to multiplex multiple bioreactors, or to develop even larger culture vessels [91,163,164]. For many academic cell manipulation techniques, particularly those involving physical or material stimulus, it will be difficult to implement them at these sorts of scales unless they can be incorporated into hollow fibres or microcarriers [165–167]. Early consideration of the steps required to move sequentially along this pathway of scale will help to avoid pauses during later clinical trials, as has been the case with some commercial products [168].

## Regulatory considerations

The regulatory pathway is also a major factor determining the speed of progress towards clinic [115]. Every cellular product is unique and determining the correct therapeutic classification is a key first step [112]. However, the distinction between transplant and cell therapy is largely determined by the level of manipulation that the cells undergo, and whether this classes as ‘substantial’, and therefore a manufacturing step. Within Europe, this is described through the 2001/83/EC Directive defining advanced therapy medicinal products (ATMPs) which includes: gene therapies, sCTMPs, and TEPs [169]. As discussed the distinction between sCTMPs and TEPs is largely down to the product's MoA [9]. Subsequent updates via the 1394/2007 ATMP regulation have amended this definition to include combination-ATMPs, where a medical device (e.g. a biomaterial) is an integral component [170]. As discussed, the FDA in the U.S.A. regulates cell therapies through their HCT/P regulations depending on the level of manipulation and intended use of the implanted material [68].

In both jurisdictions there are regulatory routes which can ease the requirements for new products and speed their passage through clinical trial. Orphan status, relating to medical conditions with small patient populations, can drastically reduce the clinical trial requirements before attaining market authorisation [171,172]. In 2017, the FDA also introduced a new regenerative medicine advanced therapy designation (RMAT) which allows for expedited trialling of new therapies which tackle life threatening conditions or meet serious unmet needs [173]. Since 2017 several MSC products have been awarded RMAT status [64].

Of course, the variety of ATMPs pose a challenge to regulators, and in many cases there are overlaps with medical device regulation if a carrier material is used. This is particularly relevant in the case of 3D bioprinted implants. Currently, the existing regulation fails to define a clear path for the manufacturing and quality control of these patient-specific treatments. In some jurisdictions (e.g. Australia) they may be completely unregulated if using autologous cells [174]. As a result, clinical trials of 3D printed ATMPs are even more scarce. Hourd *et al.* [175] reported the example of a 3D printed nasal implant which could also contain a cellular component . In this study, they examine the regulatory challenges of delivering this type of therapy into clinic. Central to this is the requirement to demonstrate GMP quality control of the implant, which by nature will be personalised to each patient.

## Conclusions

The rapid expansion of MSC research is driving an increased rate of early phase clinical trials which utilise MSCs for therapeutic purposes. However, there appears to be a bottleneck with significant attrition when moving beyond Phase II studies as relatively few treatments have reached market. In addition, it is apparent that very few current MSC therapies are utilising biomaterials or methods for manipulating cell phenotype, instead relying on the patient's body as the bioreactor to differentiate the cells. This review has taken an academic perspective, highlighting some of the key challenges when taking new MSC culture techniques towards clinical use and has highlighted many key papers which offer guidance for new researchers planning their own translational activities.

The importance of understanding therapy reimbursement has been highlighted, as cellular products can be costly to produce when considering the time and manual cell culture operations. Many high-profile commercial failures indicate that insufficient attention is being given to clinical benefit when developing these therapies. The price difference between minimally manipulated tissue products and cultured ATMPs has also been noted. However, cultured therapies appear to offer far greater control over the final product composition, offering opportunity to precisely define cell phenotype and potency.

Linked with patient benefit are the importance of *in vivo* models to demonstrate efficacy, safety and the potential for clinical benefit. Without strong *in vivo* evidence, it will be difficult to secure investment or funding for further technical development. Sub-optimal predictive capabilities of *in vivo* models are a key barrier to market which can lead to attrition for new therapeutics at later stages of development. For cellular products, it can be increasingly hard to identify suitable models for efficacy and safety testing since specific facilities and training are required for cellular products. This does, however, signal a developmental opportunity for non-animal technologies, such as humanised *in vitro* and *ex vivo* models.

In terms of manufacturing, there is the potential for the largest impact on end cost and product success. The use of allogeneic cells does offer benefits of scale but cell expansion technologies must also continue to develop. The use of novel cell manipulation technologies will only work if they can be readily up taken into GMP manufacturing and if they are compatible with promising processes such as microcarrier culture.

Although last on this list, the regulatory path has the ability to determine how long, and how much it will cost for a therapeutic to reach market. As with the other aspects of this review, early engagement with the regulatory body of interest can help to avoid a false start in terms of pre-clinical development and the manufacturing process.

## Abbreviations

ACI: autologous chondrocyte implantation
ATMP: advanced therapy medicinal products
BMP: bone morphogenetic protein
CDK: cyclin dependant kinase
CRO: contract research organisation
CXCL: C-X-C motif chemokine ligand
ECM: extracellular matrix
ERK: extracellular signal-related kinase
FAK: focal adhesion kinase
FCS: foetal calf serum
GLP: good laboratory practice
GMP: good manufacturing practice
HCT/P: human cells, tissues, and cellular and tissue-based product
HGF: hepatocyte growth factor
HLA-DR: human leukocyte antigen-DR isotype
ISCT: International Society for Cellular Therapy
MACI: matrix-induced autologous chondrocyte implantation
MoA: mode of action
MSC: mesenchymal stem/stromal cell
QALY: quality-adjusted life years
RGD: arginylglycylaspartic acid
RMAT: regenerative medicine advanced therapy designation
sCTMP: somatic cell therapy medicinal product
TEP: tissue-engineered product
TGFb1: transforming growth factor beta 1
